# K13-propeller gene polymorphisms of *Plasmodium falciparum* and the therapeutic effect of artesunate among migrant workers returning to Guangxi, China (2014–2017)

**DOI:** 10.1186/s12936-019-2984-7

**Published:** 2019-10-16

**Authors:** Jun Li, Yunliang Shi, Weiwei Zhang, Hui Yan, Kangming Lin, Shujiao Wei, Haiyan Wei, Yichao Yang, Shanping Huang, Yuxin Lu, Anxiang Ma, Jian Qin

**Affiliations:** 1 Institute of Parasitic Disease Prevention and Control, Guangxi Zhuang Autonomous Region Center for Disease Control and Prevention, Nanning, 530028 China; 2 The Peoples Hospital of Shanglin County, Nanning, 530500 China; 3 Shanglin County Center for Disease Control and Prevention, Nanning, 530500 China

**Keywords:** *Plasmodium falciparum*, Africa, K13-propeller gene, Artesunate treatment, Artemisinin resistance

## Abstract

**Background:**

The resistance of *Plasmodium falciparum* to artemisinin has been identified in Asia and some parts of Africa. The drug resistance of *P. falciparum* will be an obstacle to the successful elimination of malaria by 2025. Whole-genome sequencing of the artemisinin-resistant parasite line revealed mutations on the *k13* gene associated with drug resistance in *P. falciparum*. To understand the artemisinin resistance of the imported *P. falciparum* cases from Africa, the mutations in the *k13* gene in parasites from imported malaria cases in Guangxi Province were detected and the treatment efficiency of artesunate monotherapy was observed.

**Methods:**

DNA was extracted from 319 blood samples from migrant workers with *P. falciparum* infection who returned to their hometown in Guangxi Province from Africa between 2014 and 2017. The *k13*-*propeller* gene was amplified by nested PCR, and sequencing, gene mutation frequency and geographic difference of imported *P. falciparum* cases were analysed by comparison with the wild-type strain. Of 319 patients, 158 were *P. falciparum*-infected and were treated with intravenous injection of artesunate and were observed, including the time of asexual stage clearance and the dose of artesunate used.

**Results:**

Of the 319 *P. falciparum* samples, 12 samples had the *k13*-*propeller* mutation, and 11 point mutations were detected; 5 were non-synonymous mutations (T474I, A481T, A578S, V603E, G665S) and were not associated with artemisinin resistance. The clinical treatment observation showed that the median (IQR) dose of artesunate for peripheral blood parasite asexual stage clearance was 407.55 (360–510) mg, and the D3 parasite clearance rate was 70.25%, including the five *k13*-*propeller* mutations of *P. falciparum*. After 7 days of treatment, 98.73% of cases were cleared. Two cases were treated with artemisinin for 8 days with a 960-mg dose to completely clear the asexual parasite, but they did not have a mutation in the *k13* gene.

**Conclusions:**

Five mutations of the *k13*-*propeller* gene in 319 *P. falciparum* samples from patients returning from Africa were identified. The frequency of the *k13*-*propeller* mutants was low, and the mutations were not strongly associated with artemisinin resistance. The median (IQR) dose of artesunate monotherapy in actual clinical treatment to remove asexual parasite stages was 407.55 (360–510) mg, equivalent to D3–D4. Some *P. falciparum* cases without a *k13*-*propeller* mutation showed obvious delayed clearance of the parasite from peripheral blood.

*Trial registration* The diagnosis of malaria and the treatment of malaria-infected patients are the routine work of Centres for Disease Control and Prevention. Information on the patients was conveyed with the patient’s approval, and the research aim, methods, risks and benefits of the study were explained in detail to the patients

## Background

The resistance of *Plasmodium falciparum* to artemisinin and its derivatives has attracted worldwide attention. The WHO reported a decreased sensitivity of *P. falciparum* to artemisinin and warned of the possibility and danger of its resistance in 2005 [1]. The resistance of *P. falciparum* to artemisinin was first reported in Thailand and the Cambodian border area of the Greater Mekong River Basin in Southeast Asia [2, 3], and within 10 years, the epidemic of artemisinin-resistant strains spread rapidly to the whole Mekong River Basin, resulting in great challenges to the prevention and treatment of malaria [4, 5].

Recent studies have confirmed that chromosome 13 is associated with *P. falciparum* drug resistance [6–8]. Studies found that the C580Y, R539T, Y493H, M476I, and F446I mutations of the k13 gene are closely related to the resistance of artemisinin [9, 10]. However, the mutations of the *k13* gene vary in different regions. For example, in Cambodia, Vietnam, Laos, the C580Y mutation is the dominant mutation (has the highest prevalence at 50%); at the Thailand/Myanmar/China border, F446I is dominant, and there are a small number of C580Y mutations; and the most common mutation in Africa is A578S [11–13]. Although there is not sufficient evidence to suggest that resistance to artemisinin derivatives of *P. falciparum* originating in the Mekong River Basin has spread to Africa, South America, Oceania and East and Central Asia [14–16], the decrease in sensitivity to artemisinin in several cases recorded in Africa is not related to the mutation of *k13* but to the severe infection in children [17–19]. However, *P. falciparum* imported into China from Equatorial Guinea in Africa has been found to be resistant to artemisinin [20] in vitro.

As a province of China, Guangxi has had no local cases of malaria reported since 2003 and is now in the early stage of malaria elimination [21]. In recent years, a large number of workers in Guangxi have travelled to Africa, mostly to engage in gold washing; the returning workers can bring malaria back to China [22]. Approximately 300 imported malaria cases are reported annually in Guangxi; more than 90% were imported from the African continent, and more than 60% were *P. falciparum*. Therefore, monitoring of the imported malaria, identification of anti-malaria drug resistance and case management are the main challenges in the present stage of malaria control, especially the monitoring and identification of drug resistance, which plays an important role in elimination. In this study, the *P. falciparum k13* gene mutation characteristics of Chinese migrants from Africa and the effects of artesunate in practical clinical treatment were studied and observed, exploring the drug resistance of imported malaria from Africa and providing useful guidance for actual clinical treatment.

## Methods

### Sample collection and *Plasmodium falciparum* detection

Blood samples were obtained from 319 migrant workers with uncomplicated *P. falciparum* infection who returned from Africa to Guangxi Province between 2014 and 2017. Blood was collected before treatment, and thick and thin blood smear films were prepared and stained with 3% Giemsa for 40 min. The diagnosis of the microscopic examination was conducted by an experienced technician. The DNA was extracted from the blood samples using the QIAamp DNA Mini Kit (QIAGEN Inc., Germany) according to the manufacturer’s instructions and stored at − 20 °C for use in PCR assays. The diagnosis of *P. falciparum* was performed by nested PCR according to the Chinese industry standards (WS259-2015). Briefly, the first round amplified the *Plasmodium* spp. genus with the universal primers rPLU5/rPLU6 (rPLU5 5′-CCTGTTGTTGCCTTAAACTTC-3′ rPLU6 5′-TTAAAATTGTTGCAGTTAAAACG-3′). Then, nested PCR was performed with the *P. falciparum* specific primers rFAL1/rFAL2 (rFAL15′-TTAAACTGGTTTGGGAAAACCAAATATATT-3′ rFAL2 5′-ACACAATGAACTCAATCATGACTACCCGTC-3′).

### *k13*-*propeller* gene amplification and sequencing

The *k13*-*propeller* gene of *P. falciparum* was amplified by the nested PCR method as previously described [23]. Briefly, first-round PCR was performed in a 20 μl reaction volume containing 1.0 μl each of the forward and reverse primers AF (GCCTTGTTGAAAGAAGCAGAA) and AR (CGCCATTTTCTCCTCCTGTA) (10 μmol/l) and 17 μl of KAPA 2G Robust Mix (JianLian Gene Technology Co., Ltd., Beijing, China) and 1.0 μl of DNA template. Nested PCR was performed using a DNA Applied Biosystems 9700 thermal cycler (Life Technologies, Singapore). The PCR conditions were 95 °C for 15 min, followed by 30 cycles at 94 °C for 1 min, 59 °C for 1.5 min, and 72 °C for 2 min, and a final extension at 72 °C for 10 min. One microliter of the first-round product was used as the template in the second-round amplification, with the same cycling conditions as the first round. The amplified products were sent to XiangYin Biotechnology Co. Ltd. (Shanghai, China) and sequenced using an ABI platform (3730XL).

### Sequencing alignments and data analysis

The sequences were analysed by Mutation Surveyor 4.1 to remove the false positive and (or) false negative mutant sites and aligned to reference *Pf*3D7_1343700 (http://www.plasmodb.org) using Mega7 [24]. The *k13*-*propeller* allele frequency was also calculated.

### Clinical observations

To observe the clearance of *P. falciparum* treatment with artesunate monotherapy, 158 of the 319 *P. falciparum*-infected patients in Shanglin County People’s Hospital between 2014 and 2017 were enrolled and analysed. The axillary temperature in these patients was more than 37.5 °C, the parasitaemia was between 1000 and 200,000/μl, and anti-malarial drugs were not taken at least 3 months before hospitalization. The asexual parasite clearance time and the dosage of artesunate were observed.

Artesunate (Gulin Pharmaceutical Co., China) at 2.4 mg/kg body weight was injected intravenously (IV bolus). Since all the patients were adults, they were given 120 mg daily according to the drug instructions. Thick and thin smears were taken on day 0 (before treatment) and during subsequent prescribed follow-up on days 1, 2, 3, 4, and 5 until asexual parasite clearance was achieved. Patients were treated with oral dihydroartemisinin piperaquine (KBN, Zhejiang Pharmaceutical Co., China) for 2 days after the asexual parasite was undetectable and discharged from the hospital. Blood films were made using 3% Giemsa staining for 40 min and were independently read by two experienced microscopists to identify and calculate the number of parasites. The average of the two counts was taken as the parasite density. If the results of examination of malaria parasites between the two microscopists were inconsistent, nested PCR was used for final determination.

## Results

### Patient information

A total of 319 samples were collected from migrant workers from 23 countries in Africa during 2014–2017. Most of the patients suffered from fever, chills, sweating, headache, and diarrhoea, and few had serious complications. Of them, 42 were collected in 2014, 120 were obtained in 2015, 89 were taken in 2016, and 68 were collected in 2017. Of the 319 patients, the majority had returned from West Africa (46.71%, 149/319) and Central Africa (40.44%, 129/319), and the percentages of workers from South Africa and East Africa were only 10.66% and 2.19%, respectively (Table 1).

**Table 1:** *Plasmodium falciparum* sample geographic origin, year of collection and distribution of K13-propeller polymorphisms

Region	Country	Year of collection				Total	Mutation no
		2014	2015	2016	2017		
South Africa						34	
	Mozambique	2	1	2	0	5	
	Zambia	1	0	1	1	3	
	Angola	7	7	8	2	24	
	Malawi	0	0	1	0	1	
	Madagascar	0	1	0	0	1	
East Africa						7	
	Ethiopia	0	0	0	1	1	
	Uganda	0	0	3	1	4	1
	Tanzania	1	0	0	0	1	
	South Sudan	0	1	0	0	1	
West Africa						149	
	Mali	0	1	0	0	1	
	Ivory Coast	2	8	0	7	17	
	Liberia	1	0	2	8	11	
	Ghana	10	37	29	19	95	6
	Sierra Leone	4	3	5	0	12	1
	Guinea	1	2	1	0	4	1
	Nigeria	3	2	4	0	9	
Central Africa						129	
	Chad	0	0	2	0	2	1
	Gabon	0	1	2	0	3	
	Congo, DRC	1	2	8	3	14	1
	Congo	0	3	7	6	16	
	Cameroon	9	51	10	17	87	1
	Equatorial Guinea	0	0	3	1	4	
	Central Africa	0	0	1	2	3	
Total		42	120	89	68	319	12

### K13-propeller point mutations and distribution

A 744 bp fragment was amplified from all the samples by nested PCR, and sequencing was successfully performed using 319 *P. falciparum* samples collected from 23 countries in Africa during 2014–2017 in Guangxi. Except a sequence from Democratic Republic of the Congo found two mutated points (1/319), the other 11 sequences were single nucleotide polymorphisms (SNPs) 3.45% (11/319), 11 point mutations were identified, and the 12 mutated samples were distributed in 7 countries including Ghana, the Republic of Guinea, Uganda, Democratic Republic of the Congo, Chad, Sierra Leone, and Cameroon; with the exception of Ghana, the other countries had only one mutated sample (Table 2). In the 11 mutated points, only two point mutations were identified as two sequence mutations, whereas the other mutations were single sequence mutations. In the 12 samples, only a sequence from the Congo had two point mutations, and the others were a single point mutation. In the 11 point mutations, 6 were synonymous, and 5 were non-synonymous mutations (T474I, A481T, A578S, V603E, G665S). Of these, the mutated sequences were from Ghana (T474I, A481T, A578S, G665S), Cameroon (A578S), and Sierra Leone (V603E) (Table 2).

**Table 2:** Polymorphisms observed in the K13-propeller gene of *Plasmodium falciparum*

Codon position	Mutations	Amino acid reference	Nucleotide reference	Amino acid mutation	Nucleotide mutation	Prevalence of mutation (%)	Years	Country	Number
469	Synonymous	C	TGC	C	TGT	n = 1 (3.13%)	2016	Congo, DRC	C-87
474	Non-synonymous	T	ACA	I	ATA	n = 1 (3.13%)	2015	Ghana	B114
478	Synonymous	T	ACC	T	ACG	n = 2 (6.26%)	2014/2016	Guinea**/**Uganda	A-9/C-53
481	Non-synonymous	A	GCT	T	ACT	n = 1(3.13%)	2016	Ghana	C-76
502	Synonymous	Y	TAT	Y	TAC	n = 1 (3.13%)	2016	Congo, DRC	C-87
557	Synonymous	A	GCA	A	GCT	n = 1 (3.13%)	2015	Ghana	B-107
578	Non-synonymous	A	GCT	S	TCT	n = 2 (6.26%)	2016/2017	Cameroon**/**Ghana	C-28/E8
589	Synonymous	V	GTC	V	GTG	n = 1 (3.13%)		Chad	C-68
603	Non-synonymous	V	GTA	E	GAA	n = 1 (3.13%)	2016	Sierra Leone	C-14
610	Synonymous	K	AAA	K	AAG	n = 1 (3.13%)	2016	Ghana	C-37
665	Non-synonymous	G	GGT	S	AGT	n = 1 (3.13%)	2015	Ghana	B-22

Of 12 mutated samples, 8 were from West Africa (Ghana, Sierra Leone, Guinea) and 3 were from Central Africa (Chad, Democratic Republic of the Congo, Cameroon), one was from East Africa (Uganda), and no mutations were detected in the 34 samples from South Africa. Most of the returning workers were from Ghana (95) and Cameroon (87) (Table 1). In Ghana, 6 DNA mutated samples were detected, with a mutation prevalence of up to 6.32% (6/95), while in Cameroon, only one mutant sequence was detected, with a mutation prevalence of 1.15% (1/87). Two mutated sequences from the Democratic Republic of the Congo were detected. One DNA mutation was detected in the K13-propeller gene in Uganda, Chad, the Republic of Guinea, Uganda, and Sierra Leone (Table 2).

### Observations regarding clearance delay

**Fig. 1:**
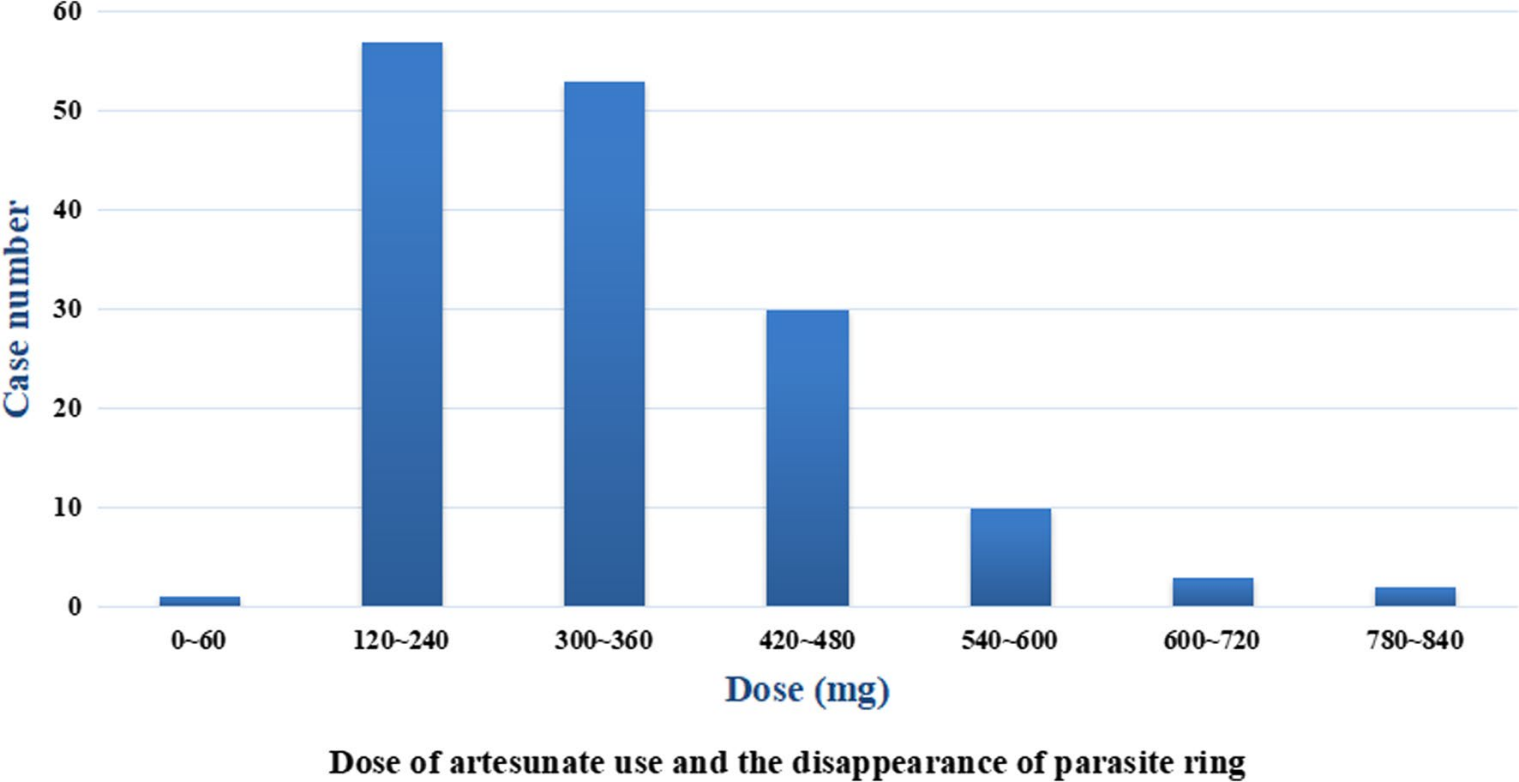
Dose of artesunate use and the disappearance of the parasite ring. Cumulative artesunate use: D1 (120 mg) had 0 cases of clearance; D2 (240 mg, n = 58) had 36.71% clearance; D3 (360 mg, n = 111) had 70.25% clearance; D4 (480 mg, n = 141) had 89.24% clearance; D5 (600 mg, n = 151) had 95.57% clearance; D6 (720 mg, n = 154) had 97.46% clearance; D7 (840 mg, n = 156) had 98.73% clearance; and D8 (960 mg, n = 158) had 100% clearance

The median (IQR) dose of intravenous artesunate to remove asexual blood parasites from the peripheral was 407.55 (360–510) mg in 158 cases. Artesunate dosage and efficacy were D1 (120 mg), 0 cases of clearance; D2 (240 mg, n = 58), 36.71%; D3 (360 mg, n = 111), 70.25%; D4 (480 mg, n = 141), 89.24%; D5 (600 mg, n = 151), 95.57%; D6 (720 mg, n = 154), 97.47%; D7 (840 mg, n = 156), 98.73%; and D8 (960 mg, n = 158), 100% (Fig. 1). The sequenced T474I (n = 1), A481T (n = 1), A578S (n = 1), V603E (n = 1), and G665S (n = 1) mutations were all cleared within D3, with no delay observed. Nine (C-87, B114, A-9, C-53, C-87, B-107, C-28, E8, C-37, B-22 Table 2) of the 158 patients who were observed the artesunate treatment detected the mutation of *P. falciparum k13* gene, of the nine mutated samples, 8 point mutations were identified, 3 points are non-synonymous and the other 5 are synonymous. In 9 mutated *P. falciparum* had not found the delayed clearance, the parasite were cleared by artesunate within day 4.

## Discussion

In this study, 5 amino acid mutations of the *k13*-*propeller* genes from 319 *P. falciparum* were found, of which 4 mutations (T474I, A481T, A578S) had been reported previously [9, 13, 22, 25–27] and had been detected in Africa [14, 25–27]. The A578S mutation has been commonly observed in Africa [14–16, 19, 23, 26, 28, 29] but was also detected in Myanmar, Thailand, India, and Bangladesh. Notably, the C580Y, R539T and Y493H substitutions that were associated with delayed parasite clearance in Southeast Asia were not observed, nor was the M476I mutation that was selected in vitro in a Tanzanian parasite [9] or the M579I mutant that was first reported in Africa artemisinin-resistant *P. falciparum* in a Chinese worker returning from Equatorial Guinea [20]. Ariey et al. [9] reported the 2 polymorphism sites T474I and A481A from Ghana. In the present study, 2 mutant sites, V603E and G665S, were detected, which had not yet been described. According to the clinical observations, these 2 mutants were not related to the clearance delay.

In the 319 cases of *P. falciparum,* 6 mutations were synonymous, of which C469C, T478T, A557A, V589V, and K610K had been reported in African countries, but through the review of other research studies, revealing that some of these mutations are non-synonymous mutations. For example, the amino acid 478 in Cameroon [23, 26] and in the present study (from Ghana) was a synonymous mutation (T478T), whereas in Mali, it was non-synonymous (T478P) [26]; amino acid 589 from the Democratic Republic of Congo [14] and from Chad (in this study) was a synonymous mutation (V589 V) but was non-synonymous in Mali and Equatorial Guinea (V589I) [25, 26]; amino acid 610 from Ghana (in this study) was a synonymous mutation (K610 K), but the mutation from Nigeria was non-synonymous K610R [27]; and amino acid 557 from Ghana (in this study) was a synonymous mutation, but the mutation from the Democratic Republic of Congo was non-synonymous (A557S) [14, 23, 25]. All of these findings indicated that the mutations in different African countries vary, and the mutations are diverse, even when the prevalence of the mutation is very low.

In the present study, the clinical data of the enrolled patients and all the *P. falciparum* patient data and treatment information were recorded and analysed, and the mutant *P. falciparum* cases were confirmed, but the clearance delay of the parasite was not detected. The WHO’s Global Plan for Artemisinin Resistance Containment prioritizes the monitoring of artemisinin-based combination therapy (ACT) efficacy to detect resistance, and this is most credibly quantified using clinical efficacy data. The *k13*-*propeller* gene amplification associated with the clinical data analysis could better determine the ACT efficacy and the artemisinin or artemisinin-derivative resistance. Artemisinin resistance has not yet been clearly defined. The slow clearance of malaria in patients treated with artemisinin or ACT is considered to be an external manifestation of resistance [30]. *Plasmodium falciparum* ring-stage susceptibility assay (RSA) in vitro [31] and half-life counts in vivo [32, 33] have been widely used to monitor the resistance of *P. falciparum* to artemisinin. *Plasmodium* still exists in the peripheral blood after treatment with the recommended dosage for 3 days, which is considered drug resistance.

In the clinical observation, the median dose of artesunate needed to remove the parasite was 407.55 mg (D3–D4). This result was similar to that of work by Dondorp et al. [3] conducted in Pailin, Cambodia, but significantly different from the results that Byakika-Kibwika et al. [34] observed in children in Uganda. Among 158 patients, 29.7% (n = 47) of blood smear asexual parasites were still positive after 3 days of treatment, and 2 even needed 960 mg (D8) to completely clear the parasite. Although many factors could affect the drug efficacy without *K13* gene mutations, including host immunity, biological characteristics of *P. falciparum* and drug factors [35], the positive rate is significantly higher after D3. According to epidemiological investigations, migrant workers who travel to remote regions in Africa and return to Guangxi and are repeatedly infected with malaria cannot undergo standardized treatment. This is a potential explanation for the higher positive rate after D3. Therefore, the observation implied that the efficacy of artesunate against *P. falciparum* imported from regions in Africa is at risk of decline.

## Conclusions

*Plasmodium falciparum* resistance to ACT was not found through the observed clinical treatment, but the efficacy of artesunate is at risk of decline, and the *k13*-*propeller* gene amplification and analysis of 319 samples of *P. falciparum* from workers returning from Africa detected 11 mutation sites, including 6 synonymous mutations and 5 non-synonymous mutations. No mutation sites were associated with delayed parasite clearance.

## Data Availability

The data from this study are available on request from the corresponding author.

## Abbreviations

ACT: artemisinin-based combination therapy
RSA: ring-stage susceptibility assay
